# Prediction of the Unconfined Compressive Strength of One-Part Geopolymer-Stabilized Soil Under Acidic Erosion: Comparison of Multiple Machine Learning Models

**DOI:** 10.3390/ma19010209

**Published:** 2026-01-05

**Authors:** Jidong Zhang, Guo Hu, Junyi Zhang, Jun Wu

**Affiliations:** 1 School of Urban Rail Transportation, Shanghai University of Engineering Science, Shanghai 201620, China; m405123206@sues.edu.cn (J.Z.); m405124223@sues.edu.cn (J.Z.); 2 School of Civil Engineering, Shanghai Normal University, Shanghai 201418, China; cvewujun@shnu.edu.cn

**Keywords:** acidic environment, geopolymer, soil stabilization, compressive strength, machine learning model

## Abstract

This study employed machine learning to investigate the mechanical behavior of one-part geopolymer (OPG)-stabilized soil subjected to acid erosion. Based on the unconfined compressive strength (UCS) data of acid-eroded OPG-stabilized soil, eight machine learning models, namely, Adaptive Boosting (AdaBoost), Decision Tree (DT), Extra Trees (ET), Gradient Boosting (GB), Light Gradient Boosting Machine (LightGBM), Random Forest (RF), Support Vector Machine (SVM), and eXtreme Gradient Boosting (XGBoost), along with hyper-parameter optimization by Genetic Algorithm (GA), were used to predict the degradation of the UCS of OPG-stabilized soils under different durations of acid erosion. The results showed that GA-SVM (R^2^ = 0.9960, MAE = 0.0289) and GA-XGBoost (R^2^ = 0.9961, MAE = 0.0282) achieved the highest prediction accuracy. SHAP analysis further revealed that solution pH was the dominant factor influencing UCS, followed by the FA/GGBFS ratio, acid-erosion duration, and finally, acid type. The 2D PDP combined with SEM images showed that the microstructure of samples eroded by HNO_3_ was marginally denser than that of samples eroded by H_2_SO_4_, yielding a slightly higher UCS. At an FA/GGBFS ratio of 0.25, abundant silica and hydration products formed a dense matrix and markedly improved acid resistance. Further increases in FA content reduced hydration products and caused a sharp drop in UCS. Extending the erosion period from 0 to 120 days and decreasing the pH from 4 to 2 enlarged the pore network and diminished hydration products, resulting in the greatest UCS reduction. The results of the study provide a new idea for applying the ML model in geoengineering to predict the UCS performance of geopolymer-stabilized soils under acidic erosion.

## 1. Introduction

Coastal regions, serving as globally significant economic and demographic hubs, are subject to environmental problems caused by acid-rain erosion and to geotechnical challenges arising from soft-ground instability, thus necessitating systematic reinforcement of their weak foundations. However, the high-pollution and high-energy-consumption characteristics of traditional cement binders are seriously against the concept of global sustainable development [1]. Geopolymer, as a low-carbon and environmentally friendly cementitious material [2], has attracted much attention due to its low energy consumption and low carbon dioxide emissions, which can reduce greenhouse gas emissions by 44–64% compared with ordinary cement [3]. The primary precursors for geopolymer are industrial by-products that are widely available, such as fly ash (FA), ground-granulated blast-furnace slag (GGBFS), and metakaolin [4,5,6,7,8]. Under alkaline activation, these precursors react to form gel phases with binding properties, which are mainly sodium aluminosilicate hydrate (N-A-S-H) in fly ash/metakaolin systems and calcium silicate hydrate (C-S-H) in slag-rich systems [9]. In addition, geopolymers exhibit excellent durability. For example, alkali-activated FA geopolymer has low permeability and self-healing capacity, giving it superior resistance to acid attack, sulfate erosion, and chloride-ion penetration compared to ordinary Portland cement [10,11,12]. Thus, geopolymers have many advantages as a soil binder over traditional cement.

However, coastal areas are not only characterized by high water content, high compressibility, and low bearing capacity [13,14,15], but are also exposed to the hazards of acid rain erosion. There is still a relative lack of research on the durability of geopolymer-stabilized soils in acidic environments (e.g., acid rain, industrial wastewater leakage, etc.). It is well known that acidic environments significantly affect the mechanical properties of stabilized soils. Among common acidic solutions, dilute HNO_3_ causes relatively mild degradation, whereas H_2_SO_4_ induces much more severe deterioration [16]. This difference is mainly attributed to the ability of Ca^2+^, K^+^, and Na^+^ in stabilized soils to form nitrates with NO_3_^−^, thus neutralizing some of the erosive effects. Thus, the micro-structure and hydration products of geopolymer-stabilized soils under acidic environment tend to be altered, which in turn affects their mechanical properties [17,18,19]. Min et al. [20] revealed that the geopolymers activated by sodium silicate were effective in stabilizing soft soils by generating hydrated gels that bind soil particles and form a dense matrix.

Traditional methods, which are time-consuming, labor-intensive, and difficult to control, often yield data with limited reproducibility and reliability, thus making it challenging to fully elucidate the complex relationship between microstructure and mechanical properties of stabilized soil. To address these limitations, machine learning (ML) techniques have been increasingly adopted. The ML models, including neural networks (NNs) [21], Random Forests (RFs) [22], Support Vector Machines (SVM) [23], and Extreme Gradient Boosting (XGBoost) [24], have been extensively utilized to predict the mechanical properties of geopolymers. For example, Ahmad et al. [25] used ML models such as Decision Tree (DT), Bagging, and Adaptive Boosting (AdaBoost) to predict the FA-based geopolymer concrete’s compressive strength, and the results showed that the Bagging model had the highest prediction accuracy with an R^2^ value of 0.97. Ahmad et al. [26] used an artificial neural network, Boosting, and AdaBoost models to predict the compressive strength of high-calcium FA-based geopolymer concrete. It was found that the Boosting model had the best performance with an R^2^ value of 0.96. Cao et al. [27] predicted the compressive strength of FA-based geopolymer concrete by using an SVM, multilayer perceptron, and XGBoost model, in which the XGBoost model had the highest prediction accuracy with an R^2^ value of 0.98. Khan et al. [28] predicted the compressive strength and acid resistance of recycled coarse aggregate concrete by using DT, RF, XGBoost, and AdaBoost models. The XGBoost model was found to perform best in compressive strength prediction with an R^2^ value of 0.995.

The majority of ML models have demonstrated remarkable proficiency in predicting the mechanical properties of geopolymers. However, their inherent black-box nature remains a significant challenge. The intricate complexity and non-linear relationships embedded within these models render their internal decision-making processes opaque and difficult to decipher. This opacity, in turn, somewhat constrains the broader generalization of these models to engineering applications. To address this limitation, researchers often employ SHapley Additive exPlanations (SHAP) and partial dependence plots (PDP) to conduct interpretable analyses of the models. For example, Elshaarawy et al. [29] used SHAP and PDP analyses to predict the compressive strength of ultra-high-performance concrete, identifying age, fiber, cement, silica fume, and high-efficiency water reducers as the key influencing factors. Alharbi [30] applied an ML model to predict soil expansion potential and used SHAP analysis to show that the plasticity index (PI) dominated the model predictions, accounting for approximately 75% of the influence, with a critical threshold identified at a PI of 55%. Mahmood et al. [31] used PDP and SHAP analyses to examine the effects of key characteristics (e.g., cement dosage, coarse aggregate dosage, and superplasticizer admixture) on the compressive strength of high-strength glass powdered concrete. Bai et al. [32] demonstrated predictive modeling of the compressive strength and splitting tensile strength of basalt fiber-reinforced concrete using a Random Forest algorithm combined with multiple hyper-parameter optimization methods. Chen et al. [33] explored the influence of input parameters on the output of unconfined compressive strength (UCS) by training a series of machine learning models and analyzing them with SHAP and PDP.

This study investigates the evolution mechanism of unconfined compressive strength (UCS) in one-part geopolymer (OPG)-stabilized soft soil under acidic erosion using interpretable machine learning (ML). Unlike conventional ML approaches primarily focused on predictive accuracy, this research emphasizes model interpretability by integrating SHAP (SHapley Additive exPlanations) and partial dependence plots (PDPs) to quantify the effects of key factors. Based on 288 experimental datasets with input features including the FA/GGBFS ratio, acid solution pH, erosion duration, and acid type, eight ML models combined with Genetic Algorithm (GA) optimization were evaluated. The GA-SVM and GA-XGBoost models demonstrated superior prediction performance. Their interpretability was then leveraged to quantitatively analyze the non-linear influences and interaction effects of various factors on UCS degradation. These data-driven insights were further validated and explained through scanning electron microscopy (SEM) observations at the microstructural level. The primary significance of this work lies in its application of interpretable ML to the complex problem of acid erosion in OPG-stabilized soils. This approach not only provides a reliable predictive tool but also bridges the gap between macroscopic performance assessment and microscopic mechanistic understanding by offering quantitative, data-driven explanations. The findings provide a scientific basis for durability design and material optimization in geotechnical engineering applications exposed to acidic environments.

## 2. Model and Methodology

### 2.1. Linear and Polynomial Regression Models

A linear model is a simple yet widely used mathematical model whose defining feature is that the dependent variable is expressed as a linear combination of the independent variables. The expression can be expressed as $y=\beta_{0}+\beta_{1}x_{1}+\beta_{2}x_{2}+\cdots +\beta_{n}x_{n}+\varepsilon$, where y is the independent variable, $x_{1},x_{2}\ldots ,x_{n}$ is the model parameter, and ε is the error term. The linear model offers the advantages of simplicity, interpretability, computational efficiency, and suitability for data exhibiting linear relationships.

Polynomial regression is a nonlinear regression technique that captures non-linear relationships by adding higher-order terms of the input variables [34,35]. Its expression is $y=\beta_{0}+\beta_{1}x+\beta_{2}x^{2}+\cdots +\beta_{n}x^{n}+\varepsilon$, where $\beta_{0},\beta_{1},\beta_{2}\ldots ,\beta_{n}$ is the dependent variable, y is the independent variable, x represents the model parameters, and ε is the error term. Polynomial regression can effectively capture non-linear trends in the data, but its flexibility makes the choice of polynomial degree crucial to avoid overfitting.

### 2.2. Decision Tree (DT)

DT is a tree-structured predictive model that can perform classification or regression by recursively partitioning the feature space. Its core idea is to split the data into increasingly pure subsets via optimal feature tests and split points [36]. DTs commonly use information gain or the Gini coefficient as splitting criteria, defined in Equations (1) and (2), respectively.

(1) $$Gain(D,A)=Ent(D)-\sum_{v\in Values(A)}\frac{\left|D_{v}\right|}{\left|D\right|}Ent(D_{v})$$

where Ent(D) is the entropy of dataset Values(A), A is all possible values of feature $D_{v}$, and $D_{v}$ is the sub-dataset where feature A takes the value v.

(2) $$Gini(t)=1-\sum_{c=1}^{C}p(c|t{)}^{2}$$

where C is the total number of categories and p(c|t) is the probability of belonging to category t at point c.

### 2.3. Random Forest (RF)

RF is an integrated learning algorithm that improves the generalization of a model by constructing multiple decision trees and combining their predictions. The core of this approach lies in increasing the diversity of the trees through a self-sampling method and feature subset selection, which reduces the variance in the model and improves the prediction performance. In RF, the training data for each decision tree is randomly drawn from the original dataset by self-sampling, allowing repetition, and this method allows for the generation of several different training subsets, each of which trains a decision tree independently [37]. The Gini coefficient, used for feature selection, is defined in Equation (2). Bagging improves prediction performance by reducing model variance; this variance reduction can be expressed by Equation (3).

(3) $$Var(\bar{X})=\frac{\sigma^{2}}{B}+\rho \sigma^{2}(1-\frac{1}{B})$$

where B is the number of trees, ρ is the correlation between trees, and $\sigma^{2}$ is the variance of a single tree.

### 2.4. Extremely Randomized Trees (ET)

ET is a variant of Random Forest that reduces model variance by injecting additional randomness into the construction of each decision tree. Unlike RT, ET selects split points completely at random instead of optimizing them [38]. ET adopts the same ensemble structure as RT but selects split points entirely at random, markedly reducing computational cost and further enhancing model diversity.

### 2.5. Adaptive Boosting (AdaBoost)

AdaBoost is a popular integrated learning algorithm that improves classification performance by iteratively training a series of weak classifiers and combining them into one strong classifier. In each iteration, AdaBoost adjusts the sample weights based on the error rate of the previous round of classifiers, causing subsequent classifiers to pay more attention to previously misclassified samples. Eventually, the predictions of all weak classifiers are weighted and combined to form a final strong classifier, where the weight of each weak classifier is inversely proportional to its classification accuracy [39]. This algorithm has been widely used in various fields such as image recognition, text categorization, bioinformatics, and fraud detection due to its adaptability, flexibility, and robustness to noise and outliers.

### 2.6. eXtreme Gradient Boosting (XGBoost)

XGBoost is an efficient gradient-boosting framework whose core idea is to iteratively add regression trees to optimize an objective function composed of a loss term and a regularization term. The latter penalizes tree depth and the minimum sum of instance weights in each node, effectively preventing overfitting. The formula is expressed as in Equation (4).

(4) $$L(\theta )=\sum_{i=1}^{n}l_{1}(y_{i},{\hat{y}}_{i})+\sum_{k=1}^{t}l_{2}(f_{k})$$

in which, the first term $l_{1}(y_{i},{\hat{y}}_{i})$ is a loss function that calculates the difference between the model’s predicted value ${\hat{y}}_{i}$ and the true value $y_{i}$, aiming to measure the model’s predictive accuracy, and the second term $\sum_{k=1}^{t}l_{2}(f_{k})$ is a regularization term that controls the complexity of each tree $f_{k}$, including the depth of the tree and the weights of the leaf nodes, to prevent the model from overfitting.

### 2.7. Light Gradient Boosting Machine (LightGBM)

LightGBM is a highly efficient gradient-boosting framework that employs a leaf-wise growth strategy combined with a histogram algorithm to discretize continuous features, thereby significantly accelerating training and reducing memory footprint. Its objective function is similar to XGBoost, but the tree-building strategy differs: LightGBM always splits the leaf with the largest gain, which speeds up convergence yet may increase overfitting risk if uncontrolled.

### 2.8. Support Vector Machine (SVM)

**Figure 1:**
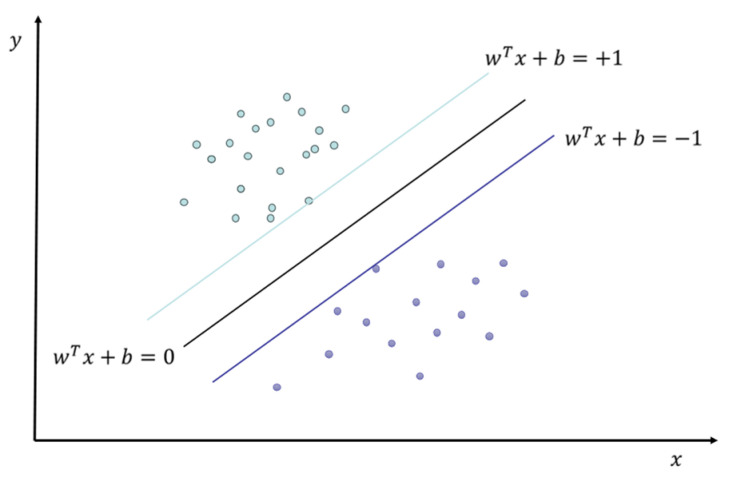
Schematic diagram of SVM classification.

SVM is a widely used supervised model for classification and regression. For classification, SVM seeks an optimal hyperplane that maximizes the geometric margin between classes. The kernel trick implicitly maps input features into a high-dimensional space, enabling linear separation without explicit coordinate computation and efficiently handling non-linear problems [40]. When data are not perfectly separable, soft-margin SVM introduces slack variables and a regularization term to balance margin size and training errors, thus mitigating overfitting [41]. Model training is typically solved via convex quadratic programming. A schematic diagram of the support vector machine classification is shown in Figure 1.

### 2.9. Genetic Algorithm (GA)

GA begins with an initial population in which each individual (chromosome) encodes a potential solution, typically as a binary string or other suitable representation [42]. A fitness function assesses every individual; those with higher fitness gain a greater probability of selection for reproduction. Selection strategies include roulette-wheel and tournament selection. During crossover, which is often regarded as the primary exploration mechanism, pairs of parents exchange gene segments to create offspring, using methods such as single-point, multi-point, or uniform crossover [43]. Mutation then randomly flips genes with a small probability, thereby injecting new genetic material and helping the population escape local optima while enhancing global search capability.

(5) $$F(x)=\left\{\begin{array}{ll} \;\;\;f(x),Maximization & \\ -f(x),Minimization & \end{array}\right.$$

The fitness function F(x) is used to assess the strengths and weaknesses of an individual, and its form depends on the specific problem. For example, in a maximization problem, the fitness function can be the objective function itself or its positive correlation function.

To assess the quality of the solution, the selection probability is determined by the fitness and is calculated as shown in Equation (6).

(6) $$P(i)=\frac{F(i)}{\sum_{j=1}^{N}F(j)}$$

where F(i) is the fitness value of individual i and N is the population size.

The relevant formula for single-point crossover is expressed in Equation (7).

(7) $$C_{1}=P_{1}[1:k]+P_{2}[k+1:n],C_{2}=P_{2}[1:k]+P_{1}[k+1:n]$$

where n is the chromosome length, $P_{1}$ and $P_{2}$ are the parent individuals, which are the two individuals in the population selected to participate in the crossover operation, and k is a randomly selected position used to determine the segmentation point of the two parent individuals based on the segmentation. $C_{1}$ denotes the first offspring individual, and $C_{2}$ notes the second offspring individual.

### 2.10. SHapley Additive exPlanations (SHAP)

SHAP is a widely-used model-interpretability framework that quantifies each feature’s contribution to an individual prediction by borrowing Shapley values from cooperative game theory. SHAP values are model-agnostic and yield both local and global explanations that consistently identify the most influential features. They can be computed exactly or via efficient approximation algorithms such as TreeSHAP and DeepSHAP [44,45,46]. The SHAP library further streamlines calculation and visualization, making model decisions transparent and trustworthy. The exact Shapley value is given in Equation (8).

(8) $$\varphi_{i}=\sum_{S\subseteq N\setminus \{i\}}\frac{|S|!\;(n-|S|-1)!}{n!}[f(S\cup \{i\})-f(S)]$$

### 2.11. Partial Dependence Plot (PDP)

The PDP is a widely used model-interpretation tool that visualizes the average effect of individual features on model predictions [47]. In complex models, PDP curves reveal the global relationship between a feature and the target while holding all other features fixed. This relationship is formalized by the partial dependence function (PDF) shown in Equation (9).

(9) $$PDF_{j}(x_{j})=E_{x_{-j}}[F(x_{j},x_{-j})]$$

where F is the predictive function of the model, $x_{j}$ are specific features that we are concerned about, $x_{-j}$ indicates all other features, and $E_{x-j}$ indicates the expected value for all other features, usually approximated by averaging over the dataset. PDP analyses can be extended from single features to two-dimensional (2D) combinations, generating 2D PDP surfaces that reveal feature interactions. By encoding predicted values with height and a color gradient on the 2D plot, these surfaces make model behavior more transparent and easier to interpret, thereby deepening understanding and enhancing overall interpretability.

### 2.12. Scanning Electron Microscopy (SEM)

Scanning electron microscopy (SEM) imaging employs a high-energy electron beam (typically 1–30 kV) to scan the sample surface point by point, stimulating secondary electrons (SEs) and backscattered electrons (BSEs). Signals are collected by detectors and simultaneously mapped into two-dimensional images. Owing to the extremely short wavelength of electrons, SEM achieves (≤1 nm) and an exceptionally large depth of field. This enables the direct visualization of microstructural features such as surface topography, grain size, pores, or cracks without requiring complex transparent sample preparation. By adjusting the accelerating voltage, working distance (WD), and detector type (SE/BSE), the spatial resolution, compositional contrast, and three-dimensionality of the image can be flexibly controlled. This makes SEM the preferred method for obtaining microstructural information in fields such as materials science, biology, and geosciences [48].

This paper will extract microscopic features from the aforementioned SEM high-resolution images and introduce SHAP (SHapley Additive Explanations) values to conduct pixel/sample-level interpretability analysis of machine learning model predictions. This establishes a transparent mapping relationship between “microscopic morphology and model decision-making”.

## 3. Methodology

### 3.1. Framework

**Figure 2:**
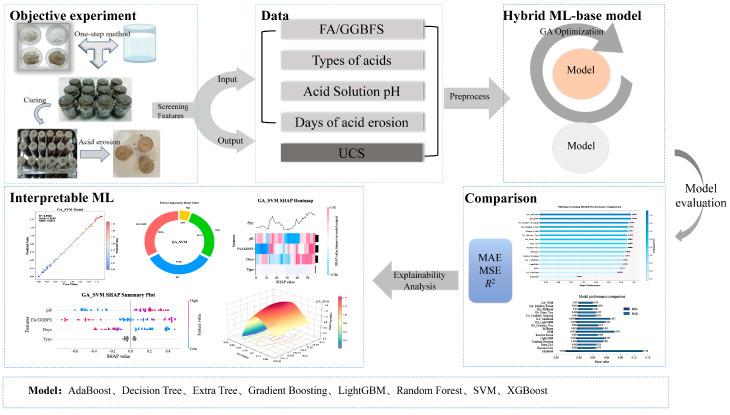
Framework of the current study.

The framework of this study was divided into 5 main parts as shown, which included objective experiments, data processing, hybrid machine learning model construction and training, model comparison, and interpretable machine learning. The framework of this study is illustrated in Figure 2.

The first part was to obtain data through experiments, which served as the source of the entire model training data. It is noted that the experimental data were obtained from the related tests conducted by our research group [20,49]. The second part was the data preprocessing phase. In this study, a multi-dimensional preprocessing procedure was systematically implemented to ensure data quality: first, categorical variables in the dataset were numerically converted using One-Hot Encoding to enable recognition of non-numerical features by machine learning models; second, a small amount of missing data (missing rate < 1%) was handled via mean imputation to avoid model training biases caused by data gaps; finally, all variables were normalized across each dimension to reduce potential biases introduced by measurement errors—specifically, min–max scaling was adopted to standardize all variables to the interval [0, 1], and the transformation is given by Equation (10). The third part focused on model development, in which 80% of the preprocessed data were allocated to the training set to construct and train 16 machine learning regressors: AdaBoost, Decision Tree, Extra Trees, Gradient Boosting, LightGBM, Random Forest, XGBoost, and SVM, together with their Genetic Algorithm-optimized variants. The fourth part employed the remaining 20% as the test set and evaluated all models using three performance metrics. The two top-performing models were then subjected to SHAP- and PDP-based interpretability analysis to elucidate their prediction mechanisms and validate feasibility.

(10) $$Norm(x)=\frac{x-\min\left(x\right)}{\max\left(x\right)-\min\left(x\right)}$$

where x is the original data, min(x) is the minimum value in the dataset, and max(x) is the maximum value in the dataset.

### 3.2. Summary of Experimental Materials and Methods

This study establishes a rigorous experimental foundation for investigating the performance of one-part geopolymer-stabilized soft soil under acidic erosion. The following section details the raw materials utilized and the standardized protocol employed for sample preparation, which is crucial for generating the high-quality dataset used in subsequent machine learning modeling.

1. Raw Materials and Their Characteristics

The physical and chemical properties of all raw materials were thoroughly characterized. The key features are summarized in Table 1 to provide a clear and concise overview.

2. Standardized Sample Preparation Protocol: The “One-Part” Method

The “one-part” or “just add water” method was adopted to prepare the geopolymer-stabilized soil samples, simulating field construction conditions. This standardized protocol ensures the reproducibility and consistency of the samples, which is paramount for generating reliable data. The procedure consists of the following critical steps:

**Step 1:** Dry mixing of precursors. GGFBS, FA, and solid NaOH were dry-mixed homogeneously according to the designed ratios (e.g., specific FA/GGBFS ratios).

**Step 2:** Geopolymer paste preparation. A specified amount of water was added to the dry mixture and stirred to form a homogeneous geopolymer paste.

**Step 3:** Soil–paste Mixing. The paste was poured into the remolded soil (prepared at a predetermined water content) and mixed rapidly using a mechanical mixer. The mixing time was strictly controlled within 5 min to prevent premature setting of the geopolymer.

**Step 4:** Sample molding. The mixture was compacted into a mold (inner walls coated with Vaseline) in three layers. Each layer was tamped approximately 15 times to remove entrapped air, forming a cylindrical specimen with a height of 8 cm and a base area of 12 cm^2^.

**Step 5:** Sealing and curing. The top surface of the specimen was sealed with plastic film. The entire preparation process, from raw material mixing to molding, was strictly completed within 45 min to prevent moisture loss.

### 3.3. Data Collection

**Figure 3:**
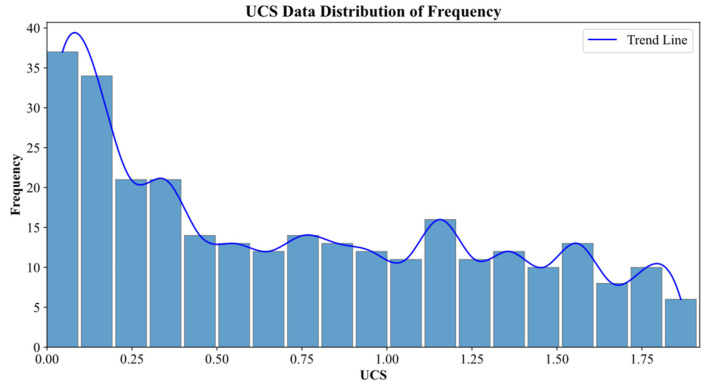
UCS data distribution map.

Data were obtained from the experimental study conducted by our research group [20,49]. In the experiment, the mass percentage of the solid silica–aluminum raw materials (FA, GGBFS, and NaOH) to dry soil was set to be 20%, the mass ratio of solid alkali exciter NaOH to silica–aluminum raw materials (FA and GGFBFS) was 0.15, and the water/cement ratio was chosen as 0.7. Meanwhile, the mass percentages of the four types of FA/GGBFS in the percussor were selected as 0/100, 10/90, 20/80, and 30/70. The initial water content for the remold soil was 50%. Table 2 reports the detailed mixing proportion of the OPG-stabilized soil sample. The prepared samples were subjected to a standard curing period of 28 days. After curing, the samples were subjected to acid erosion in HNO_3_ and H_2_SO_4_ solutions (pH 2, 4, and 6) for 30, 60, 120, and up to 240 days. The detailed experimental protocol is provided in Table 3. For model training, the input variables were FA/GGBFS ratio, solution pH, erosion duration (days), and acid type, while the target variable was the UCS of the stabilized soil. A total of 288 experimental data points were compiled [49], and their UCS distribution is presented in Figure 3.

### 3.4. Performance Characterization

Model evaluation is essential for optimization and selection. Because this study was a regression task, the mean squared error (MSE), mean absolute error (MAE), and the coefficient of determination (R^2^) were adopted as the primary metrics. Their definitions are given below.

(1) Mean Squared Error (MSE)

The mean squared error is the average of the squares between the predicted and actual values. It measures the accuracy of the model prediction.

(11) $$MSE=\frac{1}{n}\sum_{i=1}^{n}(y_{i}-\hat{y_{i}}{)}^{2}$$

(2) Mean Absolute Error (MAE)

The mean absolute error is the average of the absolute values between the predicted and actual values. It is not sensitive to outliers.

(12) $$MAE=\frac{1}{n}\sum_{i=1}^{n}|y_{i}-\hat{y_{i}}|$$

(3) R-squared (R^2^)

The R^2^ score measures the proportion of variance in the dependent variable explained by the model. It has a value range of [0, 1], and the closer the value is to 1, the better the model fits.

(13) $$R^{2}=1-\frac{\sum_{i=1}^{n}(y_{i}-\hat{y_{i}}{)}^{2}}{\sum_{i=1}^{n}(y_{i}-\bar{y}{)}^{2}}$$

### 3.5. Correlation Matrix

**Figure 4:**
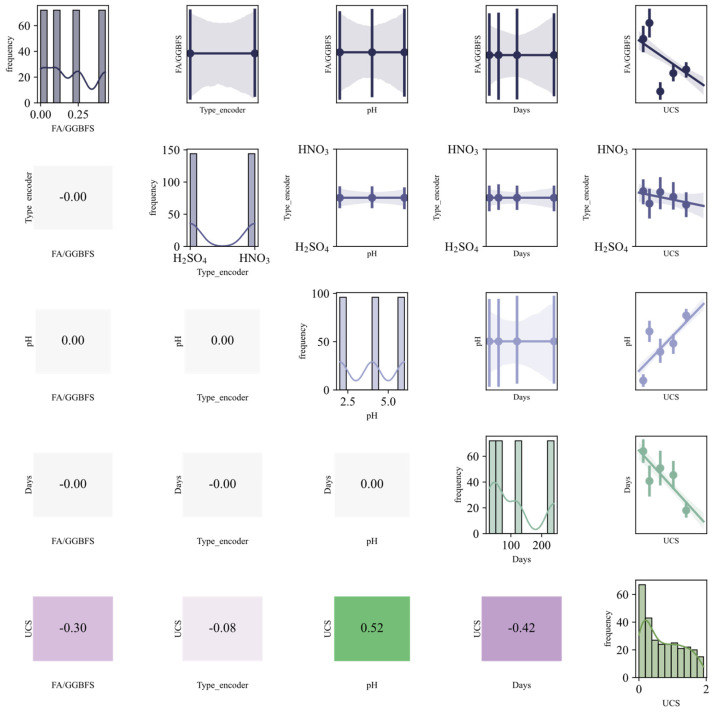
Statistical description matrix.

Multicollinearity arises when input variables are highly correlated, causing unstable model estimates [50]. To maintain model performance and stability, feature selection must therefore account for multicollinearity among predictors. Its presence is assessed via the correlation coefficient, computed as shown in Equation (14). The resulting pairwise correlations are visualized in the diagonal plot of Figure 4.

(14) $$r=\frac{\sum_{i=1}^{n}(x_{i}-\bar{x})(y_{i}-\bar{y})}{\sqrt{\sum_{i=1}^{n}(x_{i}-\bar{x}{)}^{2}}\sqrt{\sum_{i=1}^{n}(y_{i}-\bar{y}{)}^{2}}}$$

where $x_{i}$ and $y_{i}$ are the ith observation of the two variables, and $\bar{x}$ and $\bar{y}$ are the sample means of the two variables, respectively.

The statistical description matrix in Figure 4 displays variable distributions along the main diagonal: FA/GGBFS concentration centers around 0.2, confirming the optimal ratio; acidic erosion days (30–240 d) exhibit uniform distribution, ensuring comprehensive capture of extended acid exposure periods; acidic solution pH presents three peaks at 2, 4, and 6, covering strong to weak acids; UCS shows a left-skewed peak at 250–350 kPa, with low tail values indicating significant degradation in some specimens; and Type 0 and 1 isocontours ensure balanced representation of H_2_SO_4_ and HNO_3_ samples. Lower-triangle Pearson coefficients indicate that UCS negatively correlates with days (−0.63), with approximately 40% strength loss at 240 days; positively correlates with pH (+0.55), yielding an average gain of 120 kPa from pH 2 to 6; and shows |r| < 0.1 with acid type, indicating a negligible difference between HNO_3_ and H_2_SO_4_ at equivalent pH. Upper-triangle scatter-regression bands concurrently UCS decreases with increasing immersion days and increases with rising pH, remaining nearly horizontal for acid type. In summary, immersion duration and pH are the primary determinants of acid resistance, with acid type effects fully captured by pH.

## 4. Results and Discussion

### 4.1. Comparison Between Models

**Figure 5:**
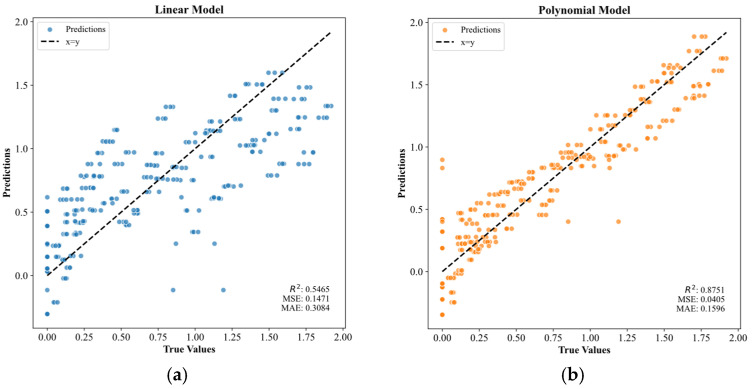
Linear model and polynomial model fitting results.

Figure 5 illustrates the relationship between the predicted results and the true values for the linear and polynomial (non-linear) models. Figure 5a shows the results of the linear model, which had an MSE of 0.1471 and a coefficient of R^2^ of 0.5465. The R^2^ value of 0.5465 indicated that the linear model had a weak ability to explain the data. Additionally, the scatter plot showed a widely dispersed distribution of points, with many points lying far from the diagonal line. This suggested a significant deviation between the model’s predictions and the actual data, implying that the relationship between the variables may not be purely linear. In contrast, Figure 5b presents the results for the polynomial model, which achieved an MSE of 0.0405 and R^2^ of 0.8751. The R^2^ value indicated that the polynomial model explained 87.5% of the variance in the data. In the scatterplot, the predicted versus observed points were markedly closer to the 1:1 diagonal, demonstrating that the polynomial model yielded more accurate predictions and suggesting a non-linear relationship within the data.

**Figure 6:**
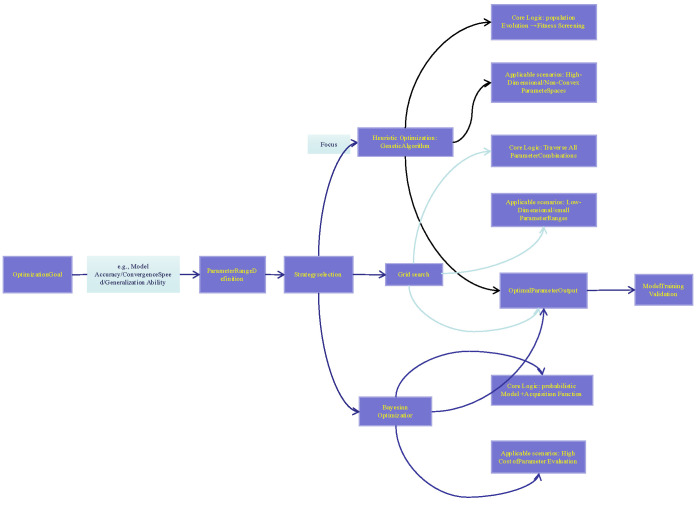
Parameter optimization flowchart.

The preceding results demonstrated that there existed a significant non-linear relationship between the variables. Although the polynomial model outperformed linear models, its representational capacity was still limited. For this reason, this study further investigated eight non-linear algorithms, namely ET, DT, RF, AdaBoost, LightGBM, GBT, SVM, and XGBoost. To ensure a fair comparison, the hyper-parameters of these algorithms were fine-tuned following the parameter optimization workflow illustrated in Figure 6. The workflow takes “model accuracy/convergence speed/generalization ability” as the optimization objectives (corresponding to the Optimization Goal module in the figure). It first defines the parameter range (the Parameter Range Definition module), and then selects optimization strategies (including grid search, Bayesian optimization, etc., corresponding to the Strategy Selection module). In this study, we focused on the Genetic Algorithm (GA)—this algorithm is suitable for high-dimensional/non-convex parameter spaces (matching the non-linear variable relationship in this study, corresponding to GA’s Applicable Scenarios in the figure), with its core logic being “population evolution + fitness screening” (corresponding to GA’s Core Logic in the figure). After the optimal parameters are obtained through this workflow (the Optimal Parameter Output module), model training and validation can be carried out, which correspond to the Model Training Validation module at the end of the figure. The model parameters optimized via GA are shown in Table 4.

It should be noted that all machine learning models were trained on 80% of the dataset and evaluated on the remaining 20%. The rationale for this split is as follows: the 80% training subset provides a sufficiently large sample size for the models to learn the inherent patterns within the data, while the 20% independent validation subset can effectively mitigate model overfitting, i.e., the phenomenon where models excessively adapt to noise in the training data. This partition enables a more objective assessment of the models’ generalization ability on unseen data, which also aligns with the “generalization ability” criterion specified in the Optimization Goal module of the workflow. Table 5 summarizes the predictive accuracy of each model in terms of R^2^, MAE, and MSE metrics. Subsequently, the model with the best performance will be selected for in-depth interpretability analysis.

**Figure 7:**
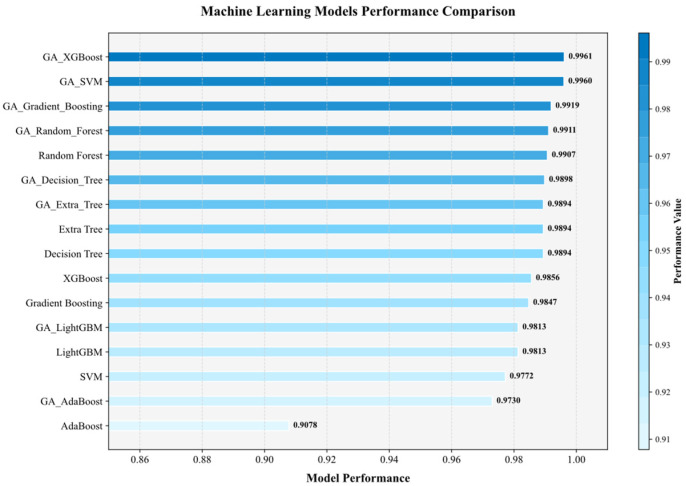
Comparison of *R*^2^ values between models.

**Figure 8:**
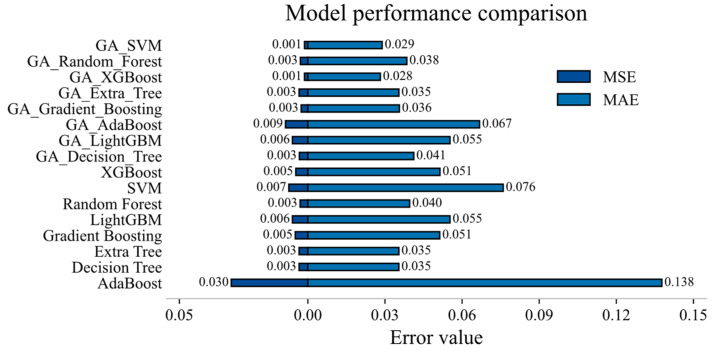
MAE and MSE between models.

Figure 7 and Figure 8 compare the R^2^, MAE, and MSE of the original and GA-optimized models. Among the unoptimized models, AdaBoost yielded the lowest R^2^, whereas RF achieved the highest. Before optimization, the R^2^ of XGBoost and SVM differed marginally. However, after GA optimization, their R^2^ values rose by 0.01 and 0.02, respectively, clearly surpassing all other models.

After comparing the MAE and MSE of the models in Figure 8, it can be found that GA-XGBoost and GA-SVM emerged as the top-performing models, benefiting both from their inherent algorithmic strengths and from the hyper-parameter tuning provided by the Genetic Algorithm. GA-XGBoost leveraged its high predictive accuracy and excellent scalability to large datasets, whereas GA-SVM capitalized on its robust handling of non-linear relationships and proven reliability in high-dimensional feature spaces.

**Figure 9:**
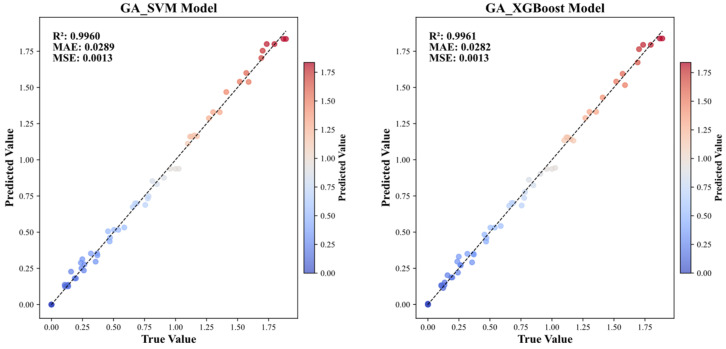
Fitting graphs for GA_SVM and GA_XGBoost.

The preceding analysis has confirmed that GA-XGBoost and GA-SVM deliver optimal performance on the acid erosion dataset. Figure 9 presents a scatter plot comparison of predicted versus actual UCS values for GA-XGBoost and GA-SVM. The scatter plots of both models are nearly identical, exhibiting only minor deviations at the extreme high and low ends. Their overall accuracy is comparable, further validating the rationale for selecting these two optimal models for subsequent SHAP/PDP interpretability analysis. Consequently, subsequent interpretability analysis shall focus exclusively on these two “high-accuracy” models. This approach ensures that SHAP/PDP analyses are grounded in reliable predictive foundations while simultaneously validating whether the “UCS degradation patterns under acidic conditions” are independent of model architecture through comparative examination of interpretative outputs. This dual verification guarantees the robustness and universality of the derived mechanistic conclusions.

### 4.2. Feature Importance

**Figure 10:**
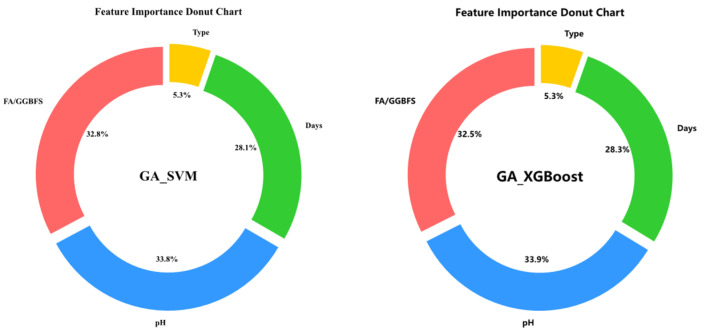
Feature importance plot for GA_XGboost and GA_SVM.

Feature-importance analysis was first employed to quantify the relative influence of each input variable on UCS. As shown in Figure 10, GA-XGBoost and GA-SVM produced virtually identical rankings: acid solution pH was the dominant predictor, followed by FA/GGBFS and days of acid erosion, whereas type of acids contributed least.

### 4.3. SHAP Analysis

In the SHAP global explanation plot, each point’s horizontal distance from zero reflected the magnitude of its impact on the model’s prediction, in which the further away, the greater the influence. The blue-to-red color scale encoded the feature value itself, that is, blue for low values and red for high values, making it easy to observe how different magnitudes of each feature shifted the predicted UCS, thus clarifying the underlying mechanisms driving the model.

**Figure 11:**
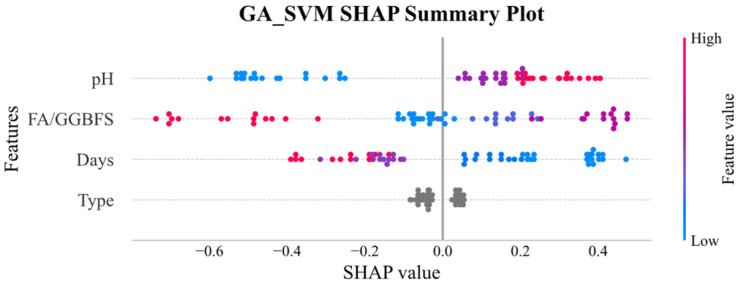
SHAP global explanation on GA_SVM.

**Figure 12:**
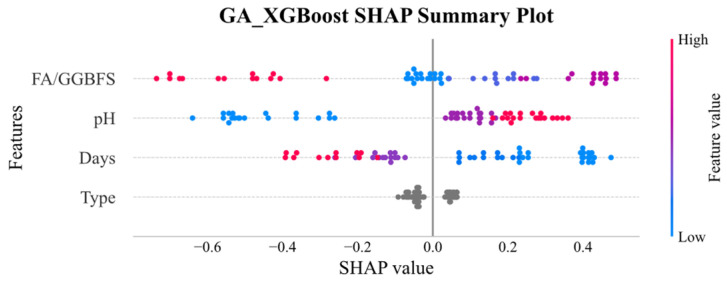
SHAP global explanation on GA_XGBoost.

Figure 11 and Figure 12 display the SHAP global explanation plots for GA-XGBoost and GA-SVM, respectively. Both models ranked the features identically: acid solution pH was the most influential, followed by FA/GGBFS, days of acid erosion, and type of acids, which had minimal impact. For acid solution pH, both models showed that lower (blue) values depressed the predicted UCS, whereas higher (red) values elevated it. This behavior might be due to the fact that lower pH corresponded to greater acidity and corrosivity, which progressively degraded the strength of the samples.

The influence of FA/GGBFS characteristics is more nuanced, yet higher values typically enhance predicted uniaxial compressive strength in both models. Acid etching duration exhibits a negative trend: longer erosion periods yield lower predicted uniaxial compressive strength, with this effect being marginally more pronounced in GA-XGBoost. Acid type exerts negligible influence in both models. Although GA-XGBoost and GA-SVM concur in feature ranking and bidirectional effects, the magnitude of specific influences diverges, reflecting their distinct internal mechanisms.

**Figure 13:**
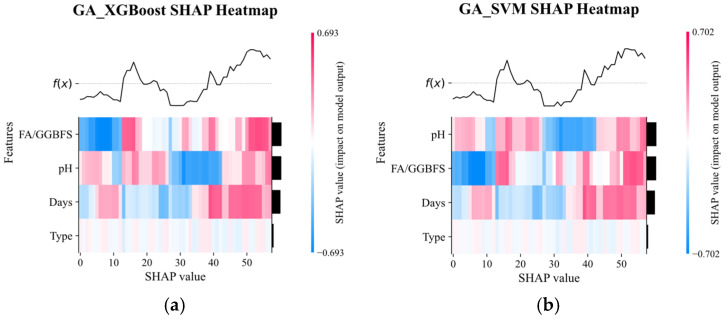
SHAP heatmap plot of GA_XGBoost and GA_SVM models.

Figure 13 displays the SHAP heat-maps for GA-XGBoost and GA-SVM. Although both models identified acid solution pH and FA/GGBFS as the dominant factors, their sensitivity to these features differed markedly. GA-XGBoost reacted more strongly to even small feature variations, reflecting its capacity to model intricate non-linear interactions. On the other hand, GA-SVM was less sensitive to the shift in individual features, and instead emphasized the identification of clear decision boundaries.

**Figure 14:**
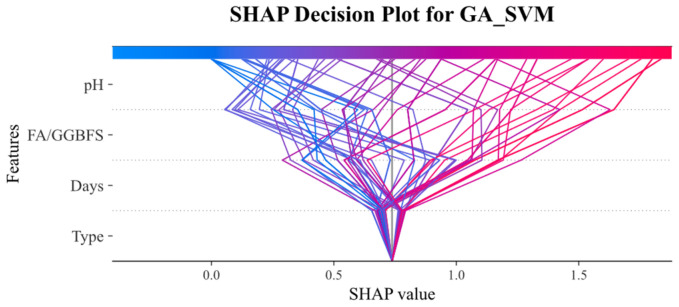
SHAP decision plot on the GA_SVM model.

**Figure 15:**
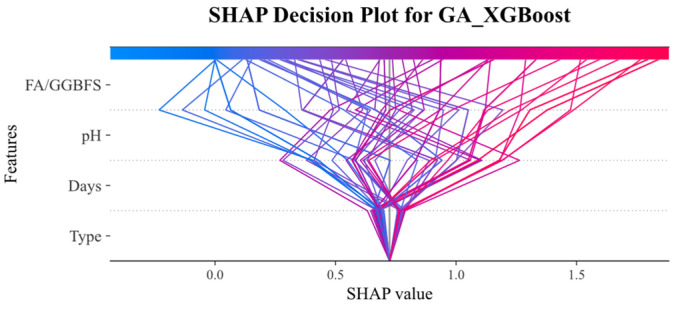
SHAP decision plot on the GA_XGBoost model.

Figure 14 and Figure 15 present SHAP decision plots for the GA_XGBoost and GA_SVM models, respectively, illustrating the contribution of individual features to the model’s output. These plots revealed that the importance of features varied between the two models. In the GA_SVM model, the pH feature was the most influential factor, whereas in the GA_XGBoost model, the FA/GGBFS feature exerted a greater impact on the prediction results.

The SHAP decision diagram visualized how the SHAP values of each feature collectively influenced the final prediction. The color transition from blue (indicating low feature values) to red (indicating high feature values) reflected the change in feature values. The wide distribution of SHAP values for the acid solution pH and FA/GGBFS features in both models suggested that changes in the feature values across samples had a significant impact on the model output.

Although SHAP values effectively revealed how individual features influenced the predictions, they provided only limited insight into the overall impact of feature interactions. To address this gap, local SHAP analysis with partial dependence plots (PDPs) should be employed. The PDP illustrated the average marginal effect of each feature across its entire range, clearly showing how changes in one or two variables shifted the model’s output while implicitly capturing interaction patterns. This global perspective can deepen the understanding of feature influence and offer actionable guidance for both model refinement and targeted feature engineering.

### 4.4. PDP Analysis

#### 4.4.1. 1D PDP Analysis

Following the application of SHAP decomposition to assess individual variable contributions, we introduced PDP analysis from a global perspective. By depicting the average model response across the full range of each feature, PDP reveals both main effects and interactions, elucidating how the model transforms inputs into predictions. Simultaneously, PDP better illustrates the dependency of each feature on the prediction target, quantifies the collective impact of key variables, and visualizes their combined effect on the UCS, thereby clarifying the model’s decision-making mechanism [51].

**Figure 16:**
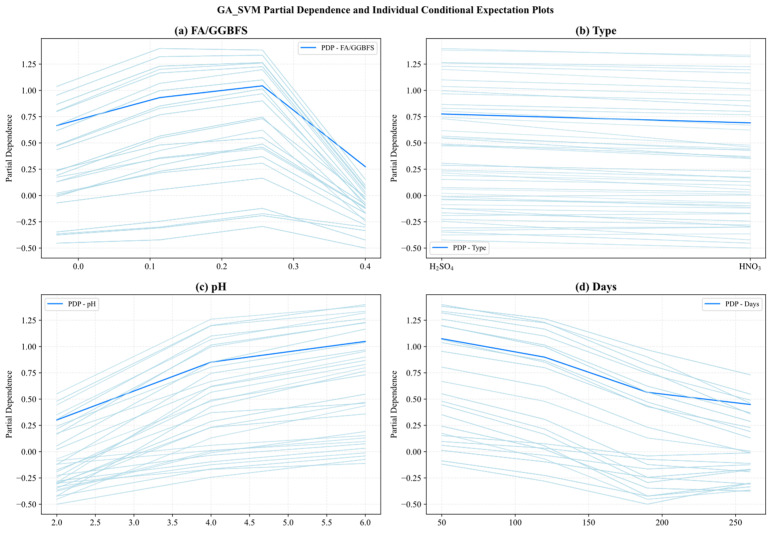
Partial dependency graph of GA_SVM.

**Figure 17:**
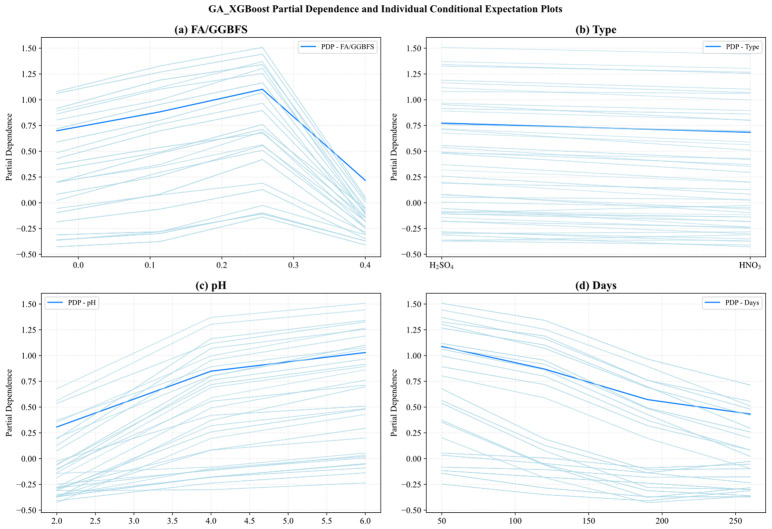
Partial dependency graph of GA_XGBoost.

Figure 16 and Figure 17 present the PDP (hidden lines) and ICE (light lines) results for GA-SVM and GA-XGBoost, respectively, across the four key features: FA/GGBFS, type of acid, acid solution pH, and days of acid erosion. Each subplot showed how varying a single feature (while holding the others fixed) affected the predicted UCS, exposing both the average marginal response (PDP) and the heterogeneity among individual samples (ICE).

Since the two models exhibited similarities in feature behavior, the main analysis herein focused on the results of Figure 16. Figure 16a reveals that the partial dependence of FA/GGBFS rose with its value, and had the peak value at FA/GGBFS of 0.25. After that, the PDP value declined. Conversely, Figure 16b shows that type of acids exerted virtually no influence, as its partial-dependence curve remained flat. This indicated that the type of acids contributed little to the prediction results under the current dataset and model settings. Figure 16c reveals a near-linear positive relationship between acid solution pH and predicted UCS. As pH increased from 2 to 6, the PDP increased steadily from 0.25 to 1.0, underscoring the detrimental effect of stronger acidity. Conversely, Figure 16d demonstrates a marked negative impact of days of acid erosion, in which the PDP decreased sharply from 1.1 to 0.5 during the first 30–60 days, then tapered off between 120 and 240 days, reflecting rapid early stage deterioration that slowed as exposure continues.

#### 4.4.2. 2D PDP Analyses in Conjunction with SEM Images

Whereas the preceding 1D PDPs (Figure 16 and Figure 17) isolated the marginal effect of each individual feature, they cannot expose interactions that occurred when features varied jointly. To overcome this limitation, the 2D PDP analysis was adopted. By simultaneously varying two predictors while holding the others constant, these 2D plots revealed non-linear and interaction effects that were otherwise hidden, providing deeper insight into how the model integrates multiple pieces of information when making its decisions.

To corroborate the interaction patterns revealed by the 2D PDP, the scanning electron microscopy (SEM) results were employed [49]. High-resolution micrographs can directly visualize microstructural changes, such as variations in porosity, hydration-product morphology, and interfacial transition zones, thereby linking the macroscopic trends predicted by the model to the underlying material response. This approach aimed to identify key feature combinations that significantly influenced model predictions and to understand their physical significance from a microstructural perspective.

**Figure 18:**
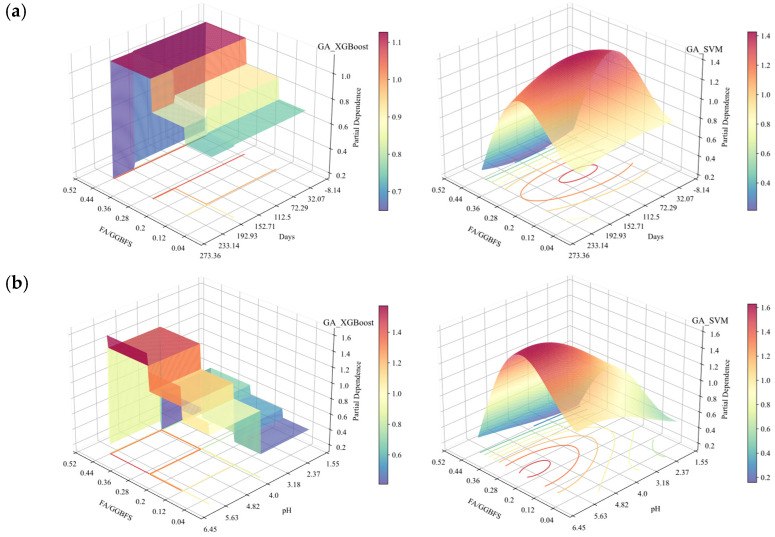

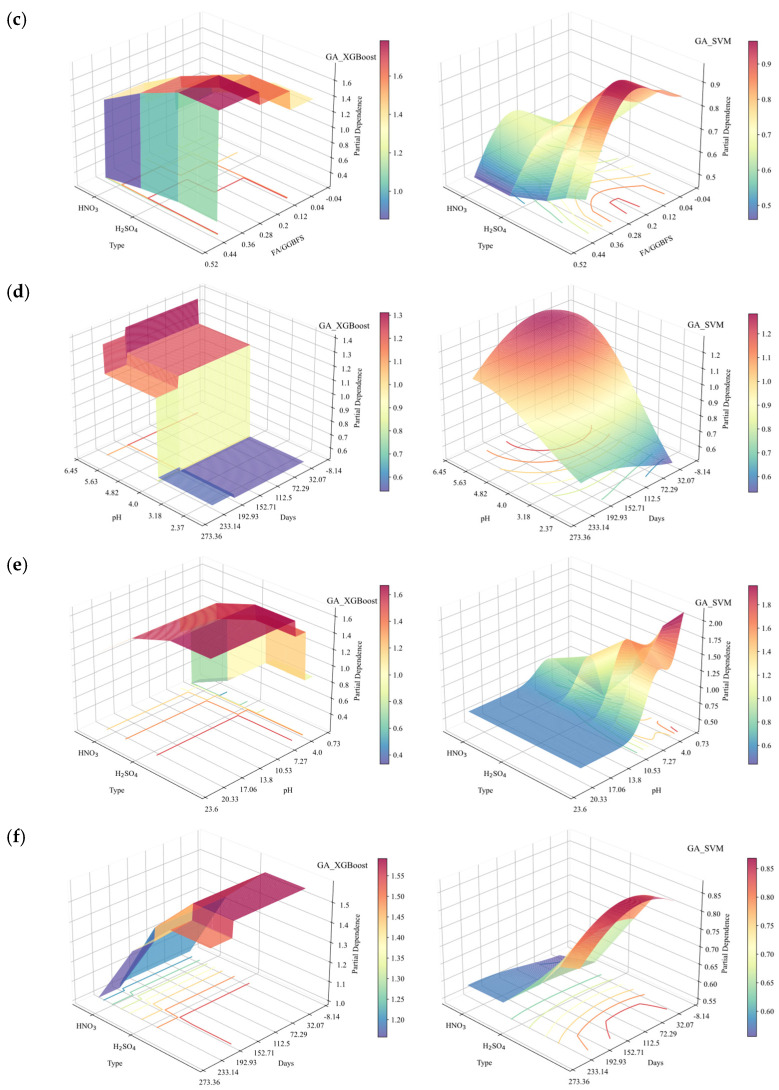
2D PDP analysis under acidic erosion (based on GA-XGBoost with a GA-SVM model): (**a**) FA/GGBFS vs. days of acid erosion; (**b**) FA/GGBFS vs. acid solution pH; (**c**) FA/GGBFS vs. types of acids; (**d**) days of acid erosion vs. acid solution pH; (**e**) acid solution pH vs. type of acids; and (**f**) days of acid erosion vs. type of acids.

**Figure 19:**
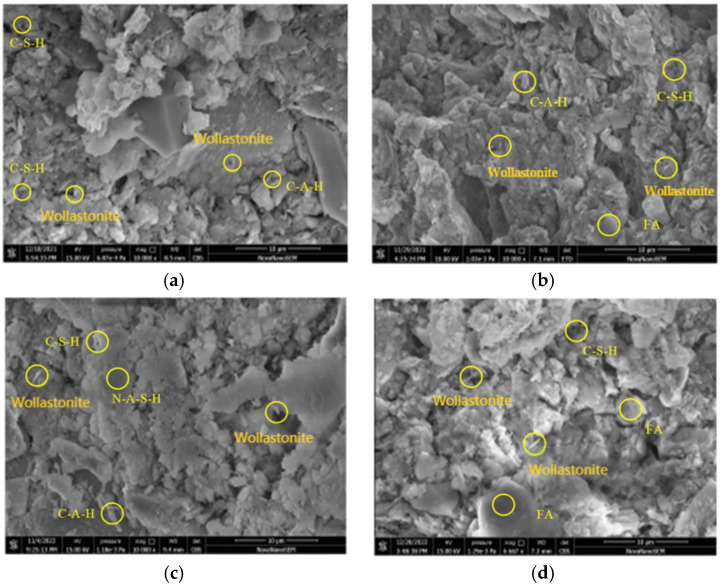
SEM results of stabilized soil with different FA/GGBFS after 60 days of erosion in H_2_SO_4_ solution at pH 4: (**a**) FA/GGBFS = 0; (**b**) FA/GGBFS = 0.11; (**c**)FA/GGBFS = 0.25; and (**d**) FA/GGBFS = 0.43.

**Figure 20:**
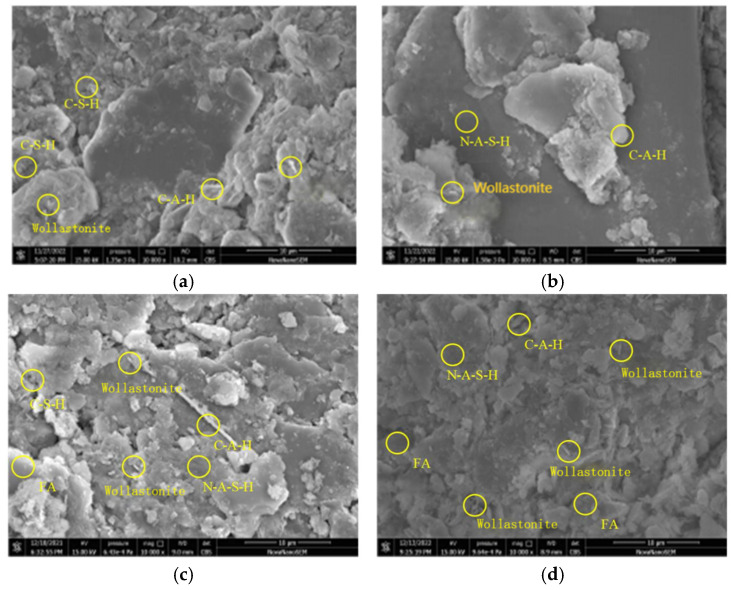
The SEM result of stabilized soil with different FA/GGBFS after 30 days of erosion in the HNO_3_ solution at a pH of 4: (**a**) FA/GGBFS = 0; (**b**) FA/GGBFS = 0.11; (**c**) FA/GGBFS = 0.25; and (**d**) FA/GGBFS = 0.43.

**Figure 21:**
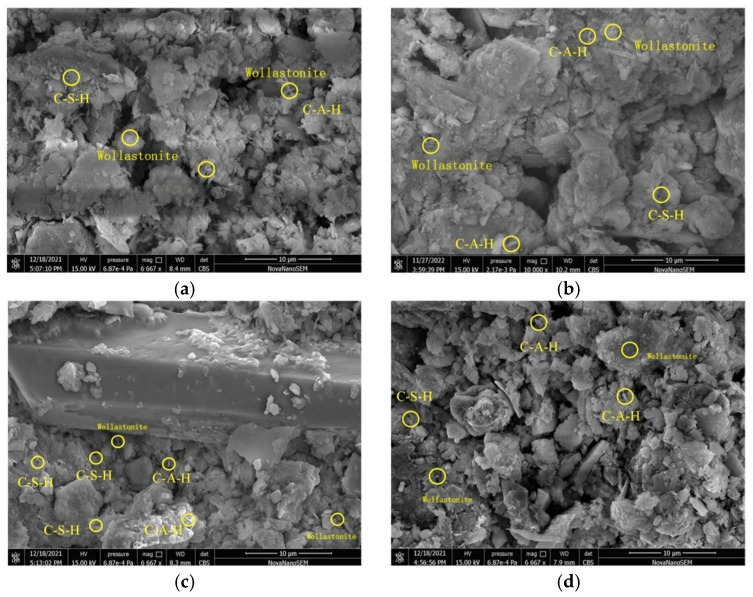
The SEM result of stabilized soil with FA/GGBFS = 0 after different days of erosion in the HNO_3_ solution at a pH of 2: (**a**) 30 days; (**b**) 60 days; (**c**) 120 days; and (**d**) 240 days.

The 2D PDP analysis for the XGBoost and SVM models is shown in Figure 18. From the figure, it was found that the general trends in the second-order interactions of the two models were essentially the same. Figure 18, Figure 19, Figure 20 and Figure 21 show the related SEM results of the samples.

From Figure 18a to c, it can be observed that the partial dependence of FA/GGBFS attained its first maximum at approximately FA/GGBFS = 0.25, with a value of ~1. This suggested that a 0.25 ratio was optimal for improving the acid resistance of OPG-stabilized soil. The same optimum was corroborated in Figure 18b, where the second partial-dependence peak occurred at FA/GGBFS = 0.25 and pH = 4, reaching ~1.2. Figure 19 presents SEM results of stabilized soil with different FA/GGBFS after 60 days of erosion in H2SO4 solution at pH 4: (a) FA/GGBFS = 0; (b) FA/GGBFS = 0.11; (c)FA/GGBFS = 0.25; and (d) FA/GGBFS = 0.43. Figure 20c presents SEM micrographs of the sample with an FA/GGBFS ratio of 0.25. Compared with the microstructures shown in Figure 19a,d and Figure 20a,d, these images revealed higher silica content, a greater abundance of hydration products (C-S-H, N-A-S-H, etc.), and a markedly denser matrix. This refined microstructure directly underpinned the observed increase in UCS.

Figure 18d shows dense contour lines between 0 and 120 days of erosion and pH value ranging from 2 to 4, denoting a steep decline in partial dependence, therefore indicating a pronounced UCS loss in this region. At a pH value of 2, the partial dependence dropped to zero after 120 days of erosion, corroborating Figure 16d and Figure 17d, in which the UCS loss rate was markedly higher from 0 to 120 days than from 120 to 240 days.

**Figure 22:**
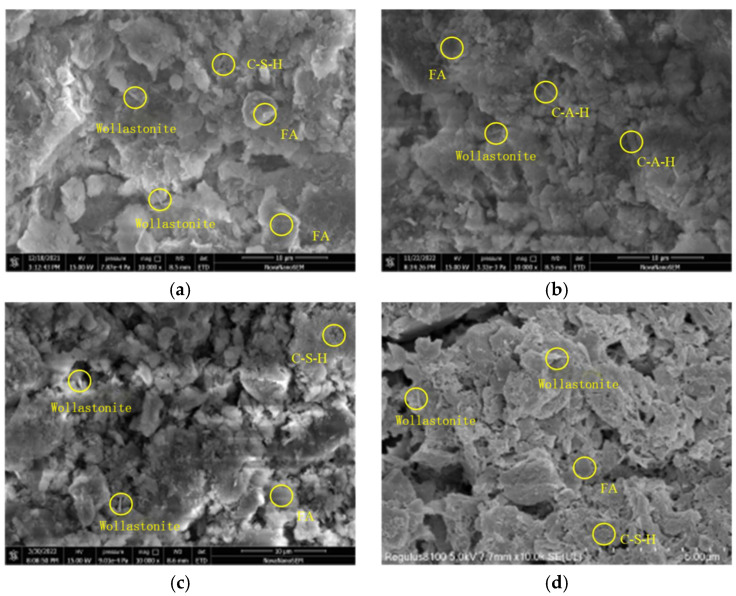
The SEM result of stabilized soil with FA/GGBFS = 0.25 after different days of erosion in the HNO_3_ solution at a pH of 2: (**a**) 30 days; (**b**) 60 days; (**c**) 120 days; and (**d**) 240 days.

Figure 21 reveals that for the sample without FA, the prolonged acid exposure progressively enlarged the pore network, in which the interparticle and hydration-product spacing widened, hydration phases diminished, and the matrix became increasingly loose. Conversely, Figure 22 shows that for the sample with an FA/GGBFS of 0.25, the excessive FA can be used to fill the pores, yielding a dense matrix that simultaneously enhanced UCS and improved acid resistance.

Additionally, the contours in Figure 18c,e,f show that the partial dependence of the sample immersed in HNO_3_ solution was slightly higher than that in H_2_SO_4_ solution under the same conditions. The SEM results in Figure 21d and Figure 22d revealed that the microstructure of the samples immersed in HNO_3_ solution for 240 days exhibited higher density. The difference was due to the formation of soluble nitrates when the sodium-hydroxide activator neutralized HNO_3_. Such a reaction lowered the free-H^+^ concentration and mitigated acid attack, whereas H_2_SO_4_ yielded less soluble products and maintained a harsher environment.

## 5. Summary

In this study, the UCS of the OPG-stabilized soils subjected to acid attack was predicted using machine learning models, including a decision tree, six ensemble learners (Random Forest, Extra-Trees, Gradient Boosting, XGBoost, LightGBM, and AdaBoost), support-vector regression (SVR) and GA-optimized hybrids thereof. The input indexes were type of acid, FA/GGBFS ratio, acid solution pH and days of acid erosion. The SHAP and PDP analyses were subsequently applied to the two best-performing GA-optimized models to elucidate how each input variable influenced the UCS of the stabilized soil. The main conclusions were as follows:

(1) The ensemble learning models, SVM, and the models optimized by the Genetic Algorithm outperformed the linear model (R^2^ = 0.5465) and the polynomial regression model (R^2^ = 0.8751) in predicting the UCS of OPG-stabilized soil under acidic environmental erosion.
(2) Genetic Algorithm optimization significantly enhanced the prediction accuracy of the ML models. Before optimization, the Random Forest and Decision Tree models already exhibited high prediction accuracy, with R^2^ values of 0.9907 and 0.9894, respectively. After optimization, GA-XGBoost slightly outperformed GA-SVM (R^2^ = 0.9961 vs. 0.9960; MAE = 0.0282 vs. 0.0289).
(3) Interpretability analyses (SHAP,1D PDP and 2D PDP) consistently identified acid solution pH as the primary determinant of UCS, followed by FA/GGBFS ratio and days of acid erosion, whereas type of acids exerted only marginal influence.
(4) Microstructural validation via SEM confirmed that an FA/GGBFS ratio of 0.25 maximized silica availability and hydration-product formation (C-(A)-S-H, N-A-S-H), yielding an optimally dense matrix and the highest acid resistance. Further increases in FA/GGBFS diluted hydration products and precipitated a sharp UCS decline. Comparative SEM also revealed marginally denser microstructures and higher UCS under HNO_3_ attack than under H_2_SO_4_, attributable to the neutralization-induced reduction in free H^+^ concentration in the nitric-acid environment.

## Figures and Tables

**Table 1:** Summary of experimental materials and their properties.

Material Name	Source/Purity	Main Chemical Composition (Mass Fraction)	Key Physical Properties and Morphology	Remarks/Purpose
Ground Granulated Blast Furnace Slag (GGBFS)	A steel plant in Suqian, Jiangsu Province	CaO (53.01%), SiO_2_ (37.29%), MgO (7.63%), Al_2_O_3_ (0.81%)	Grey-white powder; XRD shows a predominantly amorphous glassy phase; irregular, angular, flaky particle morphology.	One of the silicon–aluminum raw materials.
Fly Ash (FA)	A power plant in Wuxi, Jiangsu Province	SiO_2_ (56.38%), Al_2_O_3_ (29.46%), CaO (2.76%), TiO_2_ (2.76%)	Grey-black powder; classified as low-calcium fly ash; spherical, smooth particle morphology with high porosity.	One of the silicon–aluminum raw materials.
Sodium Hydroxide (NaOH)	Commercially available, 99% purity	-	White solid flakes.	Alkaline activator.
Test Soft Soil	Near Suzhou Creek, Putuo District, Shanghai	-	Natural water content 50.0%, liquid limit 43.3%, plastic limit 24.6, plasticity index 18.7; particle size distribution: silt (75%), clay (15%), and sand (10%).	Material to be stabilized; crushed, sieved, and dried before use.
Acidic Solutions	Yida Technology (Quanzhou) Co., Ltd.	Dilute HNO_3_, dilute H_2_SO_4_, initial concentration 1.0000 mol/L	Diluted and precisely calibrated to target pH values using a pH meter (model: pH-100; accuracy: ±0.02pH).	To simulate an acid rain erosion environment.
Water	Regular tap water	-	-	For experimental use.

**Table 2:** Mixing proportion of the OPG-stabilized soil.

Parameter	Numerical Value
(FA + GGBFS + NAOH)/Dry Soil	0.2
NAOH/(FA + GGBFS)	0.15
FA/GGBFS	0/100; 10/90; 20/80; 30/70
Water to Binder Ratio	0.7

Note: The actual calculated values for FA/GGBFS are 0, 0.11, 0.25, and 0.43, respectively.

**Table 3:** Objective experimental program.

Batch Number	FA/GGBFS	Types of Acids	pH	Days of Acid Erosion
A-1	0/100	HNO_3_	2	30
A-2			4	
A-3			6	60
A-4		H_2_SO_4_	2	120
A-5			4	240
A-6			6	
B-1	10/90	HNO_3_	2	30
B-2			4	
B-3			6	60
B-4		H_2_SO_4_	2	120
B-5			4	240
B-6			6	
C-1	20/80	HNO_3_	2	30
C-2			4	
C-3			6	60
C-4		H_2_SO_4_	2	120
C-5			4	240
C-6			6	
D-1	30/70	HNO_3_	2	30
D-2			4	
D-3			6	60
D-4		H_2_SO_4_	2	120
D-5			4	240
D-6			6	

**Table 4:** Genetic Algorithm optimization parameters and objectives.

Foundational Model Type	Optimal Model Identification Following GA Optimization	Core Optimization Parameters	Core Optimization Objectives
Decision Tree	GA Decision Tree	random_state, max_depth, min_samples_split	Balancing tree complexity and generalization capability
LightGBM	GA LightGBM	num_leaves, min_child_samples, subsample, learning_rate	Balancing LightGBM’s efficiency with predictive accuracy
AdaBoost	GA AdaBoost	learning_rate, n_estimators, base_estimator, loss	Optimize integrated weights to reduce bias
Gradient Boosting	GA Gradient Boosting	learning_rate, max_depth, subsample, min_samples_leaf	Control gradient to mitigate overfitting and enhance stability
Extra Tree	GA Extra Tree	max_features, random_state, n_estimators, max_depth	Optimize feature selection and tree structure to enhance robustness
XGBoost	GA XGBoost	max_depth, learning_rate, gamma, colsample_bytree, n_estimators	Precision control of complexity to maximize predictive accuracy
Random Forest	GA Random Forest	n_estimators, max_features, max_depth, min_samples_leaf	Optimize ensemble diversity and reduce variance
SVM	GA SVM	C, gamma, epsilon, kernel, shrinking	Balancing classification intervals and error margins to accommodate non-linear data

**Table 5:** Performance metrics of the model in predicting UCS.

Model	R^2^	MAE	MSE
AdaBoost	0.9078	0.1377	0.0298
GA_AdaBoost	0.9730	0.0668	0.0087
SVM	0.9772	0.0760	0.0074
LightGBM	0.9813	0.0553	0.0060
GA_LightGBM	0.9813	0.0553	0.0060
Gradient Boosting	0.9847	0.0513	0.0049
XGBoost	0.9856	0.0514	0.0047
Decision Tree	0.9894	0.0354	0.0034
Extra Tree	0.9894	0.0354	0.0034
GA_Extra_Tree	0.9894	0.0354	0.0034
GA_Decision_Tree	0.9898	0.0412	0.0033
Random Forest	0.9907	0.0396	0.0030
GA_Random_Forest	0.9911	0.0385	0.0029
GA_Gradient_Boosting	0.9919	0.0356	0.0026
GA_SVM	0.9960	0.0289	0.0013
GA_XGBoost	0.9961	0.0282	0.0013

## Data Availability

The original contributions presented in this study are included in the article. Further inquiries can be directed to the corresponding author.
